# Study the biomechanical performance of the membranous semicircular canal based on bionic models

**DOI:** 10.1016/j.heliyon.2022.e09480

**Published:** 2022-05-23

**Authors:** Yixiang Bian, Shien Lu, Zhi Wang, Yongbin Qin, Jialing Li, Guangming Guo, Junjie Gong, Yani Jiang

**Affiliations:** School of Mechanical Engineering, Yangzhou University, Yangzhou 225127, China

**Keywords:** Biomechanical model, Bionic semicircular canal, Membrane semicircular canal, Symmetric electrodes Metal core PVDF Fiber (SMPF)

## Abstract

A BA (bionic ampulla) was designed and fabricated using an SMPF (Symmetric electrodes Metal core PVDF Fiber) sensor, which could imitate the sensory hair cells to sense the deformation of the cupula of the BA. Based on the BA, a bionic semicircular canal with membrane semicircular canal (MBSC) and a bionic semicircular canal without membrane semicircular canal (NBSC) were designed and fabricated. The biomechanical models of the MBSC and NBSC were established. The biomechanical models were verified through the perception experiments of the MBSC and the NBSC. The results showed that the SMPF could sense the deformation of the cupula. The MBSC and NBSC could sense the angular velocity and accelerations. What's more, it was speculated that in a human body, the endolymph probably had a function of liquid mass while the membranous semicircular canal and the cupula had a function similar to a spring in the human semicircular canal.

## Highlights

• Establishment and verification of the function of the membranous semicircular canal.
• Successfully fabricated the bionic semicircular canal.
• Obtaining the function of an MBSC and an NBSC.

## 1 Introduction

The vestibular system is located in the inner ear of a human head, which includes three pairs of semicircular canals and 2 pairs of cystic organs (a utricle and a saccule) [1]. The function of the vestibular system is to sense body posture, to maintain body balance and clear vision [2]. The semicircular canals can sense the angular acceleration while the cystic organs can sense the linear acceleration of the head [3].

The 2/3 parts of the semicircular canal in the human body is an approximately circular ring, and the other part of it is an irregular ring. The largest part in the canal is the utricle, and the swelling part adjacent to it is the ampulla [4]. A semicircular canal is composed of two layers. The outer layer is the bony semicircular canal, which is full of perilymph. The inner layer is the membranous semicircular canal, which is full of endolymph [5]. The perilymph and endolymph are isolated from each other by the membranous semicircular canal [6]. There is a saddle-shaped ridge at the bottom of the ampulla in the semicircular canal, which is called a crest. A fan-shaped glial cupula is on the top of the crest, which can block the lumen of the membranous semicircular canal and prevent the convection of the endolymph in the membranous semicircular canal [7]. The bundles of the sensory hair cells grow into the cupula. When the head rotates, the wall of the semicircular canal also rotates. However, the rotation speed of the endolymph in the semicircular canal is less than that of the canal wall due to the inertia of the endolymph. Therefore, the inertia force of the endolymph acts on the glial cupula, and the concave and convex deformation occurs in the cupula. At the same time, the bundles of the sensory hair cells located in the cupula can bend and generate nerve signals, which are transmitted to the vestibular center and brain through the ascending nervous system. According to the nerve signals, the vestibular center and brain can judge the amplitude and direction of the deformation of the cupula, and thus sense the angular acceleration of the head [8], [9], [10].

Pathological changes of the vestibular system will cause the impairment of some functions of the vestibular system, and induce nystagmus, dizziness, and other symptoms [11]. To understand the working principle of the vestibular system and the cause of the vestibular dysfunction, it is necessary to establish a biomechanics model of the semicircular canal [12], [13].

It is the first time to analyze the hydrodynamic response in the semicircular canal by W. C. Van Buskirk et al. [12]. Their model was used to analyze the effect of gradient pressure on the utricle and ampulla. Their results showed that the semicircular canal could be simplified into a large damping second-order angular accelerometer. Houshyar Asadi et al. developed a continuum model to describe the macroscopic mechanical response of the inner ear in hot and cold tests [10], [14]. M. Kassemi et al. established a fluid and solid coupling finite element model for the horizontal semicircular canal, and analyzed the field distribution of the velocity and the pressure of the endolymph in hot and cold tests [15]. Cai-qin Wu et al. analyzed the interaction between the endolymph flow in the horizontal semicircular canal and the movement of the cupula using a finite element method when the human head rotated at a constant speed [16]. E. Njeugna et al. studied the dynamic characteristics of the cupula and obtained its modal shape [17]. David M. Lasker et al. obtained a group of neural responses of rat semicircular canals under sinusoidal rotation excitations [18]. V.A. Sadovnichy et al. carried out extensive research on the relationship between the nerve response of cupula and mechanical excitation using an integrated model [19]. Shen Shuang et al. established a three-dimensional semicircular canal model using the finite element method, and carried out some analysis on the endolymph-cupula coupling system, including modal shape, excitation response, and frequency characteristic [20], [21].

Those above typical biomechanical models have greatly promoted the study of the relationship between the structure and function of the human semicircular canal. However, in the above models, the membranous semicircular canal was all assumed to be rigid, and could not deform under the action of the endolymph flow. The wall of the membrane semicircular canal is a biofilm structure with a thickness of 15–50 μm, it can return to its original position after deformation due to its elasticity [22], [23]. Therefore, if the membranous semicircular canal is assumed to be rigid, there will be some resultant deviations in the established semicircular canal models [24], [25], [26]. To date, the specific function of the membrane semicircular has not been reported. It is difficult to directly display the mechanical response of the semicircular canal by existing technical methods (such as clinical imaging) because it has a complex structure and small volume and is deeply located in the human head [27]. Bionic semicircular canal, imitating the structure of human biological tissue, designed and fabricated by using artificial materials or devices, provided a new way to study biomechanics in the human semicircular canal [28], [29].

Pamela T. Bhatti et al. imitated the shape of the semicircular canal in the human body and fabricated a circular pipeline angular acceleration sensor. The pipeline was filled with liquid and equipped with a flexible capacitance sensor [30]. Charalambos M. Andreou et al. encapsulated the liquid in a micro-glass tube which was fabricated using a Micro Electro Mechanical Systems technology and pasted the piezoresistive sensor at the turning of the glass tube [31]. When the liquid flowed in the tube, the pressure of the liquid could be calculated according to the variation of the resistance value, and then the angular velocity and angular acceleration of the tube could be obtained. Nannan Chen et al. installed a piezoresistive artificial hair sensor in a pipeline, and the liquid flow in the pipeline could cause the hair cantilever beam to bend and deform [32]. Through the resistance variation of the fixed end of the cantilever beam, the deformation of the cantilever beam could be sensed. Mohammad Amin Raoufi et al. fabricated an artificial semicircular canal using a 3D printing method. They inserted a piezoresistive hair sensor into the ampulla of the artificial semicircular canal shell, injected deionized water into it, and packaged it. The results suggested their fabricated artificial semicircular canal could sense the frequency and amplitude of rotational motion [33]. Sajad Abolpour Moshizi et al. developed a flexible flow sensor based on the polyvinyl alcohol hydrogel nanocomposites, which could mimic the sensory hair cells in the human vestibular [34], [35]. After embedded the sensor in the ampulla, an artificial semicircular canal was fabricated. It can sense the stage's sinusoidal rotation [36], [37].

These models simulate the structure of the semicircular canals of the human body and have the function of sensing angular acceleration. The human semicircular canal includes membranous semicircular canal and bone semicircular canal. Therefore, the force field distributions in these models were probably different from those in human semicircular canals due to the structural differences.

In this paper, a pair of symmetric electrodes metal core polyvinylidene fluoride fiber (SMPF) sensor [38], [39], with a sensing function similar to that of the sensory hair cell, was designed to fabricate two bionic semicircular canals (BSCs), including a bionic membranous semicircular canal (MBSC) and a bionic non-membranous semicircular canal (NBSC). Through theoretical derivation, combined with the way of experiments carried out on the MBSC and NBSC, the mechanical model of the semicircular canal under rotation was established, and the specific function of the membranous semicircular canal was studied.

## 2 Materials and methods

### 2.1 Materials

The PVDF 6008 particles were purchased from Solvay Corporation. The metal core was molybdenum wire (Φ 0.11 mm, Nanjing Juxin Tungsten Molybdenum Co., Ltd.). The surface electrodes were conductive silver paste (3706, Shenzhen Sinwe Electronic Material Co., Ltd). The cupula was made of silicone rubber (E610, Shenzhen Hongye Technology Co., Ltd.). The shells of ampullas of all the models were fabricated with standard resin (A01, Shenzhen Chuangxiang 3D Technology Co., Ltd.), using a 3D printer (HoRi2300Plus, Shanghai UnionTech Co., Ltd).

### 2.2 Fabrication methods

#### 2.2.1 Fabrication of an SMPF sensor

**Figure 1:**
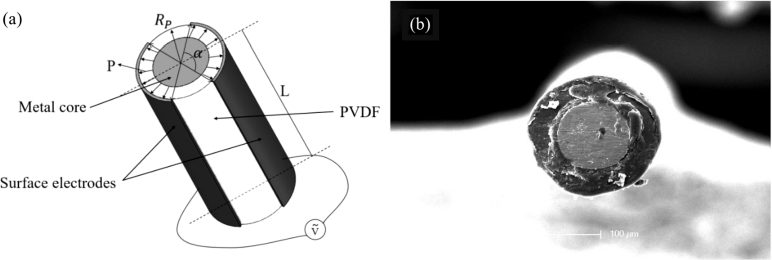
(a) A diagram of an SMPF sensor. (b) A SEM image of the SMPF.

The SMPF was fabricated according to the procedures described in our previous study [38]. Briefly, the PVDF particles were heated to 170 °C to a molten state. Then, the molten PVDF was extruded together with a tungsten wire. After air cooling, the metal core was tightly wrapped by the PVDF layer, and a fiber embryo was obtained. The fiber embryo was cut into a small size according to the requirements, and two symmetrical silver layers were coated on the longitudinal surface of the embryo as surface electrodes. Then, the fiber embryo was put into silicone oil and heated to 130 °C. At the same time, a 500 V voltage was applied to the metal core and the surface electrodes to polarize the PVDF. After the voltage was removed, an SMPF sensor was obtained. A Schematic diagram and an SEM image of the SMPF sensor are shown in Figs. 1a and 1b.

#### 2.2.2 Fabrication of BA

**Figure 2:**
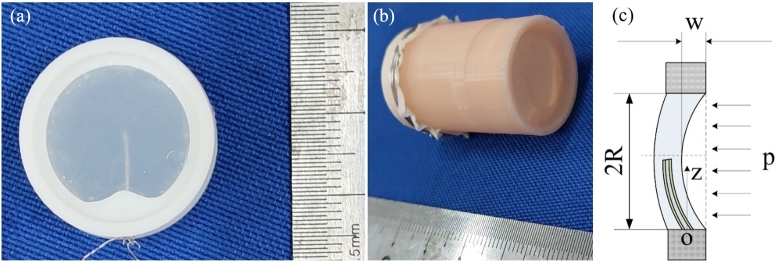
(a) A Photograph of a BA. (b) A photo of a straight tube. (c) A deformation diagram of the cupula of the BA under liquid pressure.

Based on the anatomical structure of the ampulla in the HSCC (the Human Semicircular Canal), the BA with a ratio of 10:1 to the HSCC was fabricated. The SMPF was placed in a mold, and its root was fixed on the BA shell which was fabricated using a 3D printer. Then, medical silicone rubber solution was injected into the mold, and a BA was obtained after the solution was solidified. A photograph of the BA is shown in Fig. 2a.

#### 2.2.3 Fabrication of the models

##### 2.2.3.1 Straight tube

A BA was installed on one end of a straight pipeline fabricated with resin using the 3D printing method. After the deionized water was injected into the straight pipeline, the other end of it was sealed with a rubber membrane. A photograph of the straight tube is shown in Fig. 2b.

##### 2.2.3.2 MBSC model

The structure of the BSC was similar to that of the human semicircular canal, and the ratio between them was 10:1. The real size of the human semicircular canal we used is based on the Reference [40]. The shell of the bionic bone semicircular canal was fabricated with resin by a 3D printing method, and the bionic membrane semicircular canal was fabricated with silicone rubber by mold casting method. Then the BA was fixedly connected with the shell of the bionic bone semicircular canal and the bionic membrane semicircular canal. The deionized water was separately injected into the bionic bone semicircular canal and the bionic membrane semicircular canal and an MBSC was obtained.

##### 2.2.3.3 NBSC model

The structure of the NBSC was similar to that of the MBSC, but it had not a membrane semicircular canal. The BA was installed directly in the ampulla of the bionic bony semicircular canal.

### 2.3 Establishment of the experimental system

A fixed base, a moving base, and the connecting devices such as gear and rack were fabricated using a 3D printer based on fused deposition modeling technology (HoRi2300 Plus, Beijing Huitianwei Technology Co., Ltd). The BSC model was fixed in the moving base with the components. Connected the exciter (HEV-50, Nanjing Foneng Technology Co., Ltd), moving base and rail device in turn along the moving direction of the exciter head. A rack and gear devices were fixed on the position between the exciter and the fixed base, so that linear motions provided by the exciter could be transmitted into rotational motions of the BSC models.

### 2.4 Data acquisition

The electric charges generated by the SMPF were converted into voltage using a charge amplifier (HY5853, Econ Technology Co., Ltd). Then the voltage was put into the computer through a data acquisition card (USB3202, Beijing Art Technology Co., Ltd). The displacements measured by the laser displacement sensor (LK-G5000, Keyence corporation.) were also input into the computer through the data acquisition card. Data processing was performed using Origin 8 (OriginLab, Northampton, MA).

## 3 Building theoretical models

### 3.1 The model of the relationship between the deformation of the BA and the SMPF output charges

Under the pressure of the bionic endolymph, the cupula of the BA could produce concave and convex deformation, and the SMPF embedded in it also bent and generated electric charges due to the piezoelectricity of the PVDF layer, as shown in Fig. 2c. To simplify the calculation process, the shape of the cupula was approximately regarded as a drum with a radius of *R*. One end of the SMPF was fixed on the shell of the BA, and its axial direction was also the diameter direction of the cupula in the BA. Taking the fixed end of the SMPF as the coordinate origin, the deflection curve equation of the SMPF cantilever beam under the action of a uniform surface force *p* could be described as follows [41]:

(1) $$w(z)=\frac{p}{64M}(4R^{2}z^{2}-4Rz^{3}+z^{4})$$

where *M* is the bending stiffness of the cupula, which is determined by the material characteristics and the structure size. Then the deflection at the central point of the cupula, that was, the linear displacement could be described as follows:

(2) $$h=w(R)=\frac{pR^{4}}{64M}$$

The curvature of the SMPF could be described as follows:

(3) $$\frac{1}{\rho (z)}=w^{\prime \prime }(z)=\frac{p}{64M}(8R^{2}-24Rz+12z^{2})$$

The strain at the surface electrode of the SMPF could be calculated as follows:

(4) $$S(z)=\frac{R_{p}\sin\theta }{\rho (z)}=\frac{pR_{p}\sin\theta }{64M}(8R^{2}-24Rz+12z^{2})$$

For the piezoelectric effect of the PVDF layer, electrical charges could be generated on the surface electrodes of the SMPF when the SMPF was bent. The first kind of piezoelectric equations was used to calculate the amount of electrical charges [42].

(5) $$\left[S\right]=\left[s^{E}\right]\left[T\right]+{\left[d\right]}^{T}\left[E\right]$$

(6) $$\left[D\right]=\left[d\right]\left[T\right]+\left[\varepsilon^{T}\right]\left[E\right]$$

where *D* represents the electric displacement, *S* represents the strain. $T_{0}$ is the stress and *E* is the electric field intensity. [*s*] denotes the elastic compliance coefficient matrix, [*D*] denotes the piezoelectric strain constant matrix, and [*ε*] denotes the dielectric constant matrix.

In a cylindrical coordinate system, the boundary conditions of the SMPF cantilever beam could be described as follows [43]:

(7) $$T_{\mathrm{rr}}=T_{\theta \theta }=T_{r\theta }=T_{\theta z}=T_{\mathrm{rz}}=0$$

(8) $$E_{r}=E_{\theta }=E_{z}=0$$

Substituting the boundary conditions into them, it could be obtained as follows.

(9) $$S_{\mathrm{zz}}=s_{11}^{E}T_{\mathrm{zz}}$$

(10) $$D_{r}=d_{31}T_{\mathrm{zz}}$$

Substituting the Eq. (9) into Eq. (10), then the electric displacement (the density of the charges) could be obtained.

(11) $$D_{r}=d_{31}\frac{S_{\mathrm{zz}}}{s_{11}^{E}}$$

Integrating the density of the charges on the electrode, the quantity of the charges on a single surface electrode of the SMPF could be obtained as follows:

(12) $$Q_{1}=\int_{0}^{L}\int_{\frac{\pi }{2}-\frac{\alpha }{2}}^{\frac{\pi }{2}+\frac{\alpha }{2}}D_{r}R_{p}d\theta dz$$

where $R_{p}$ is the radius of the SMPF. Submitting the Eq. (11) into Eq. (12), it could be obtained as follows:

(13) $$Q_{1}=\frac{pd_{31}R^{2}\sin\frac{\alpha }{2}}{32s_{11}^{E}D}(8R_{p}^{2}L-12R_{p}L^{2}+4L^{3})$$

where *L* is the length of the SMPF, *θ* is the central angle of the surface electrodes. Due to the symmetrical structure of the SMPF, the charges on the two electrodes exhibited equal magnitude but opposite polarity. In the sensing circuit in Fig. 1a, the quantity of the charges could be calculated as follows:

(14) $$Q=2Q_{1}=\frac{pd_{31}R^{2}\sin\frac{\alpha }{2}}{16s_{11}^{E}D}(8R_{p}^{2}L-12R_{p}L^{2}+4L^{3})$$

Submitting the Eq. (2) into Eq. (14), it could be obtained as follows:

(15) $$Q=\frac{4d_{31}\sin\frac{\alpha }{2}}{s_{11}^{E}R^{2}}(8R_{p}^{2}L-12R_{p}L^{2}+4L^{3})h$$

Setting *B* was as follows:

(16) $$B=\frac{4d_{31}\sin\frac{\alpha }{2}}{s_{11}^{E}R^{2}}(8R_{p}^{2}L-12R_{p}L^{2}+4L^{3})$$

Then the *Q* could be described as follows:

(17) Q=Bh

In other words, the SMPF sensing charges were linearly related to the linear displacement at the central point of the cupula. By measuring the amount of the SMPF output charges, the linear displacement at the central point of the cupula could be calculated, and the direction and amplitude of the concave and convex deformation of the cupula could also be obtained by further calculation.

### 3.2 The mechanical model of the MBSC and NBSC

**Figure 3:**
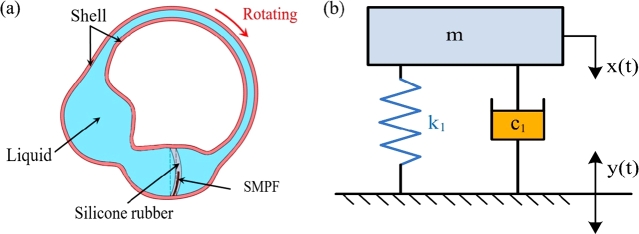
(a) When the NBSC rotates, the cupula deforms and the SMPF also bends. (b) The mechanical model of the NBSC when it rotates.

When the NBSC rotates, the cupula of the BA deforms and the SMPF also bends, as shown in Fig. 3a. To study the mechanism of the interaction between the cupula and the liquid in the NBSC, a mechanical model was established as shown in Fig. 3b. When the NBSC shell rotated, the cupula and the liquid in the NBSC also moved. For the liquid inertial force, the cupula was compressed and deformed by the liquid near it. The liquid was regarded as a mass block, and the cupula was regarded as a spring, which was elastic and produced concave and convex deformations under the action of the liquid. The case that the BSC shell was subject to sinusoidal swing was equivalent to that the basis of the mechanical model was subject to a sinusoidal linear motion. The linear displacement of the basis was assumed as follows [44]:

(18) y(t)=Asin⁡ωt

The velocity and acceleration of the basis could be calculated as follows:

(19) $$\nu =\overset{˙}{y}(t)=A\omega \sin\omega t$$

(20) $$\alpha =\overset{¨}{y}(t)=-A\omega^{2}\sin\omega t$$

The linear displacement at the central point of the cupula in the NBSC was equivalent to the deformation displacement of the spring in the mechanical model. Because the spring was closely connected with the mass block, the displacement of the spring was equal to the relative displacement of the mass block, which was defined as h(t). It could be calculated as follows:

(21) h(t)=x(t)−y(t)

Taking the mass block as a separate object, its force analysis was carried out. The mass block was affected by inertia force, damping force, and spring force, its dynamic balance equation could be described as follows [45]:

(22) $$m\overset{¨}{x}(t)+c\left[\overset{˙}{x}(t)-\overset{˙}{y}(t)\right]+k_{1}\left[x(t)-y(t)\right]=0$$

where *m* is the mass of the mass block, x(t) is the absolute displacement of the mass block, *c* is the damping coefficient, and $k_{1}$ is the stiffness coefficient of the spring.

Submitting the Eq. (21) into Eq. (22), the following Eq. could be obtained.

(23) $$mh(t)+ch(t)=-my(t)=m\omega^{2}A\sin\omega t$$

Solving the Eq. (23), the deformation of the spring could be obtained as follows:

(24) $$h(t)=\frac{\lambda^{2}A}{\sqrt{{\left(1-\lambda^{2}\right)}^{2}+{\left(2\xi \lambda \right)}^{2}}}\sin(\omega t-\varphi )$$

where *λ* is a frequency ratio, and it is defined as follows:

(25) $$\lambda =\frac{\omega }{\omega_{n}}$$

where $\omega_{n}$ is the natural frequency without damping in the mechanical model, and it is defined as follows:

(26) $$\omega_{n}=\sqrt{\frac{k}{m}}$$

where *ξ* was defined as the damping ratio and could be calculated as follows:

(27) $$\xi =\frac{c}{2\sqrt{mk}}$$

The resonance frequency of the system was defined as follows:

(28) $$\Omega =\frac{k}{m}\sqrt{1-2\xi^{2}}$$

Submitting the Eq. (24) into Eq. (17), it could be obtained as follows:

(29) $$Q=\mathrm{Bh}(t)=\frac{\lambda^{2}\mathrm{AB}}{\sqrt{{\left(1-\lambda^{2}\right)}^{2}+{\left(2\xi \lambda \right)}^{2}}}\sin(\omega t-\varphi )$$

According to Eq. (24), when the shell of the NBSC was subject to a sinusoidal swing, the displacement at the central point of the cupula in the NBSC also exhibited a sinusoidal waveform. The swing frequency at the central point of the cupula was the same as that of the shell, and the swing amplitude was linear with that of the shell and related to the swing frequency of the shell. According to Eq. (29), the SMPF output charges exhibited a sinusoidal waveform when the shell of the NBSC was subjected to a sinusoidal swing.

**Figure 4:**
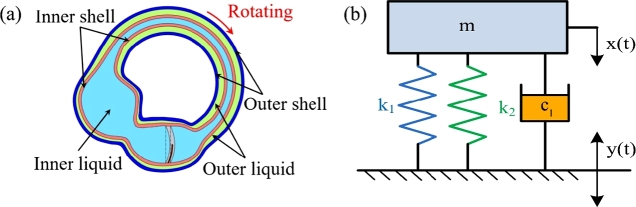
(a) When the MBSC rotates, the cupula of the BA deforms and the SMPF also bends. (b) The mechanical model of the MBSC when it rotates.

As shown in Fig. 4a, considering that the membrane of the MBSC was elastic, then it could be regarded as a spring, and its stiffness coefficient was denoted as $k_{2}$. The diagram of the mechanical model is shown in Fig. 4b. The cupula spring was parallel with the membranous labyrinthine spring, therefore, these two springs could be equivalent to a single spring, and the equivalent stiffness could be described as follows:

(30) $$K_{M}=k_{1}+k_{2}$$

When the shell of the two BSCs was subject to a simple harmonic swing, the linear displacements at the central point of the cupula respectively in the two BSCs could be described as Eq. (24). The parameters of the MBSC and NBSC were distinguished by adding subscripts *M* and *N*, respectively. Suppose that $c_{M}=c_{N}$, it could be obtained that $\Omega_{M}>\Omega_{N}$ and $h_{M}<h_{N}$ due to that $k_{M}=k_{1}+k_{2}>k_{N}=k_{1}$. This meant that the swing amplitude at the central point of the cupula in the MBSC was less than that in the NBSC when the swing frequency and swing amplitude were kept constant.

When the shells of the two BSCs were subject to an impact swing, the excitation displacement could be described as follows [46]:

(31) $$y(t)=A_{0}\sum_{n=0}^{\infty }\delta \left(t-n\frac{T_{0}}{2}\right)$$

where $A_{0}$ is the amplitude of the impact swing, δ(t) is the unit pulse function, and $T_{0}$ is the period of the impact swing.

(32) $$\overset{˙}{y}(t)=0$$

Submitting the Eq. (32) into Eq. (22), the dynamic balance equation of the mass block could be obtained as follows:

(33) $$m\overset{¨}{x}(t)+c\overset{˙}{x}(t)+k_{1}x(t)=k_{1}y(t)=k_{1}A_{0}\sum_{n=0}^{\infty }\delta \left(t-n\frac{T_{0}}{2}\right)$$

Solving the Eq. (33), the displacement of the mass block could be obtained as follows:

(34) $$x(t)=\frac{k_{1}A_{0}}{m}\frac{1}{\omega_{\mathrm{rd}}}\sum_{n=0}^{\infty }e^{-\xi \omega (t-\frac{n\pi }{\omega_{0}})}\sin\omega_{\mathrm{rd}}\left(t-\frac{n\pi }{\omega_{0}}\right)$$

The damped natural frequency of the system was defined as follows:

(35) $$\omega_{\mathrm{rd}}=\omega_{n}\sqrt{1-\xi^{2}}$$

Then the relative displacement h(t) of the mass block can be calculated as follows:

(36) $$h(t)=x(t)-y(t)=\;\frac{k_{1}A_{0}}{m}\frac{1}{\omega_{\mathrm{rd}}}\sum_{n=0}^{\infty }e^{-\xi \omega (t-\frac{n\pi }{\omega_{0}})}\sin\omega_{\mathrm{rd}}\left(t-\frac{n\pi }{\omega_{0}}\right)-A_{0}\sum_{n=0}^{\infty }\delta \left(t-n\frac{T_{0}}{2}\right)$$

where y(t) was an impact displacement, therefore, y(t) was equal to zero under the condition that t>0.

Then the h(t) could be described as follows:

(37) $$h(t)=\frac{k_{1}A_{0}}{m}\frac{1}{\omega_{\mathrm{rd}}}\sum_{n=0}^{\infty }e^{-\xi \omega (t-\frac{n\pi }{\omega_{0}})}\sin\omega_{\mathrm{rd}}\left(t-\frac{n\pi }{\omega_{0}}\right)$$

Submitting the Eq. (37) into Eq. (17), the SMPF output charges could be calculated as follows:

(38) $$Q=\mathrm{Bh}(t)=B\frac{k_{1}A_{0}}{m}\frac{1}{\omega_{\mathrm{rd}}}\sum_{n=0}^{\infty }e^{-\xi \omega (t-\frac{n\pi }{\omega_{0}})}\sin\omega_{\mathrm{rd}}\left(t-\frac{n\pi }{\omega_{0}}\right)$$

Based on Eq. (36) and (37), it could be known that the displacements at the central point of the cupula in the two BSCs exhibited gradually decaying sinusoidal waveforms when the shells of the two BSC were under the action of an impact force. And the output charges of the SMPFs in the two BSCs also exhibited a gradually decaying sinusoidal waveform, and their amplitudes were linear with the excitation amplitude of the shells of the two BSCs. Similarly, when the shells of the two BSCs were subjected to an impact vibration, the electrical signal of the MBSC was significantly smaller than that of the NBSC.

## 4 Results and discussions

### 4.1 The ability of the SMPF to sense the deformation of the BA

**Figure 5:**
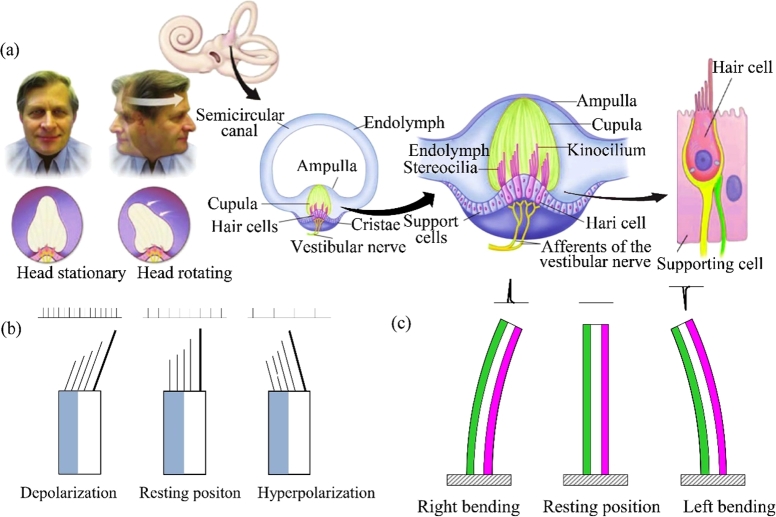
(a) Diagram of the human semicircular canal [49]. (b) Perception of the sensory hair cell based on the tilt of its own cilia. (c) A diagram of the SMPF to sense its deformation direction.

In the HSCC, sensory hair cells are the sensing organs, and their cilia are embedded in the cupula, as shown in Fig. 5a. The cilia can keep upright in a static state, as shown in Fig. 5b. Under head accelerations, the hair cells experience depolarization and hyperpolarization due to the incline direction of the cilia, the same deformation direction of the cupula. Therefore, the direction and amplitude of the deformation of the cupula can be sensed by the hair cells [47]. A diagram of the SMPF sensing function is shown in Fig. 5c. When the SMPF bends, electric charges can be generated due to the piezoelectric effect of the PVDF. The direction and amplitude of the SMPF bend deformation can be obtained according to the polarity and amount of the electric charges. Therefore, in theory, the function of the SMPF was similar to that of the hair cell, which is that they both can sense the directions and amplitudes of the bend.

**Figure 6:**
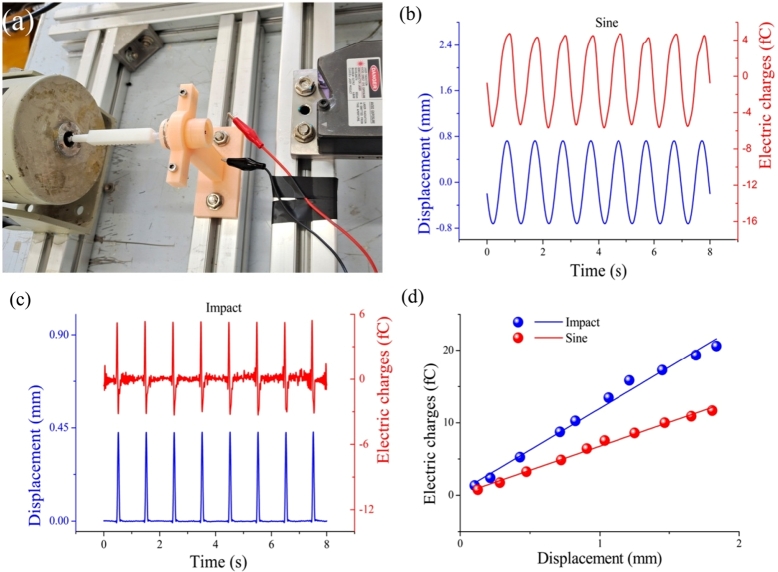
(a) An experimental system used to verify SMPF's ability to sense deformation of cupula in a BA. (b) The output electric charges of the SMPF when sinusoidal vibration occurs at the central point of the cupula of a BA. (c) The output electric charges of the SMPF when impact vibration occurs at the central point of the cupula of a BA. (d) The relationship between the SMPF sensor and the deformation of the cupula of a BA.

To study the ability of the SMPF to perceive the concave and convex deformation of the BA, a straight tube experimental system was built, as shown in Fig. 6a. In this system, one side of the BA bore the pressure of the deionized water, the other side of it was exposed to the air. An electromagnetic exciter was used to push the other end of the BA. The cupula underwent concave and convex deformation, and the SMPF in the cupula was also deformed simultaneously.

As shown in Fig. 6b, when the BA was subjected to a sinusoidal oscillation with a frequency of 1 Hz and amplitude of 0.465 mm, the displacement at the central point of the cupula exhibited a sinusoidal waveform. The waveform, frequency, and phase of the SMPF output charges were the same as those of the cupula displacement. As shown in Fig. 6c, when the BA was under an impact oscillation, the displacement at the central point of the cupula also exhibited an impact waveform. The waveform, frequency, and phase of the SMPF output charges were the same as those of the cupula displacement. In addition, the quantity of the charges rapidly decayed to a minimum after it reached a maximum, and then slowly returned to the zero position, which was due to the inherent characteristics of piezoelectric materials. So, the conclusion was that the waveform, frequency, and phase of the maximum value of the displacement at the central point of the cupula could be inferred according to the SMPF output charges. The relation between the SMPF output electric charge amplitude and the cupula central point amplitudes is shown in Fig. 6d. It can be seen that the displacement amplitude is linear with the electrical charge amplitude. In addition, as shown in Fig. 6d, the amplitude of the SMPF electric charge under the impact oscillation pattern was larger than that under the sinusoidal oscillation pattern at the same oscillation amplitude. These experimental results verified the mathematical model in Eq. (17), which was that the waveform, frequency, phase, and amplitude of the displacement at the central point of the cupula could be inferred by measuring the SMPF output electric charges.

### 4.2 The ability of the MBSC and NBSC to sense the angular acceleration

**Figure 7:**
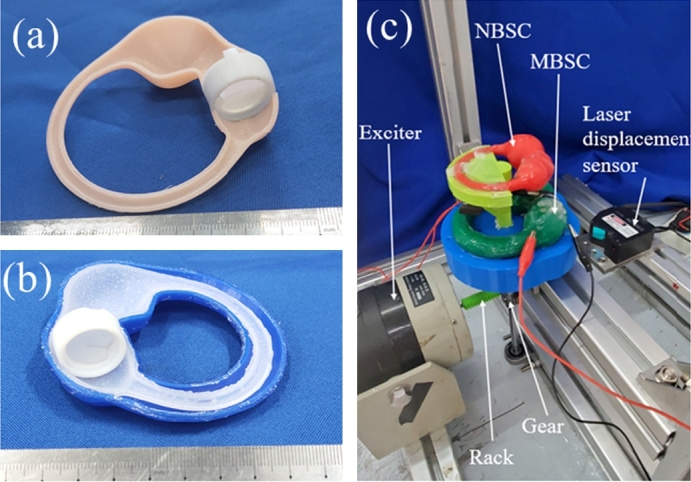
(a) A sectional photograph of the NBSC with complete BA. (b) A sectional photograph of MBSC with complete BA. (c) Angular acceleration sensing experiment system of the NBSC and the MBSC.

Sectional photographs of NBSC and MBSC with complete BA are shown in Figs. 7a and 7b. To verify the theoretical models of the NBSC and the MBSC, the experimental system was established as shown in Fig. 7c. As it is well known, each single semicircular canal in the human body is basically in one plane, and the slender part of a semicircular canal is almost arc-shaped. Similarly, either the NBSC or the MBSC model in this paper was in one plane, and also had a standard arc shape in its slender part. The straight line through the central point of the arc and perpendicular to the plane was defined as a central line. Then the MBSC and the NBSC models were horizontally placed on different floors, with their central lines coinciding with each other. The exciter was used to drive the MBSC and the NBSC models to oscillate through a rack and pinion system. Then the MBSC and the NBSC models have the same angle displacement, angular velocity, angular acceleration, and phase. The displacements of the two model shells were measured using a laser displacement sensor, and the angular displacements of the MBSC and the NBSC models could be obtained by further calculation.

**Figure 8:**
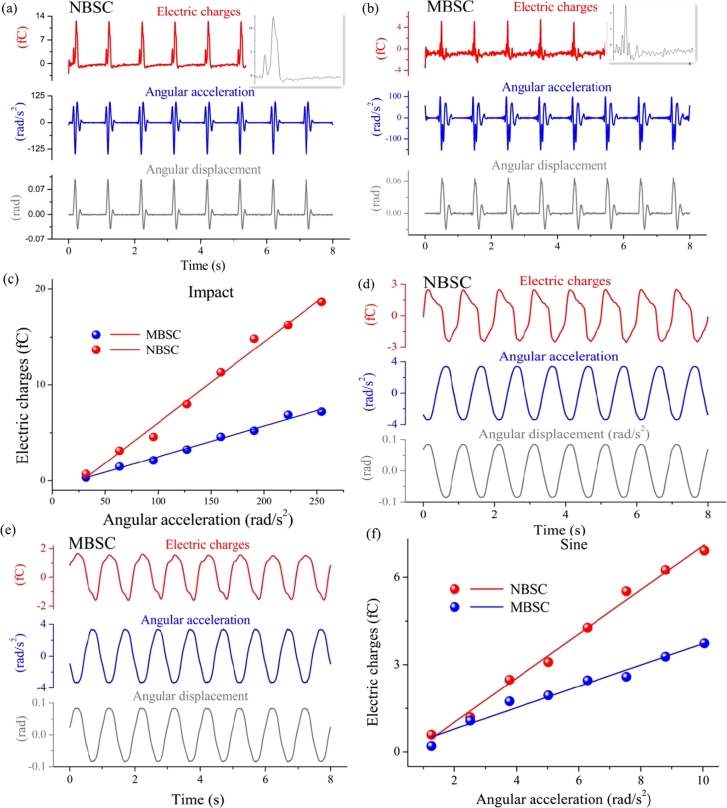
The experimental results of the angular acceleration of NBSC and MBSC. (a) The results of the NBSC's impact swing experiment include a partially enlarged view of the electric charges. (b) The results of the MBSC's impact swing experiment include a partially enlarged view of the electric charges. (c) The relationship between the deformation of the cupula and the impact angular acceleration of the NBSC and the MBSC. (d) The results of the NBSC's sinusoidal swing experiment. (e) The results of the MBSC's sinusoidal swing experiment. (f) The relationship between the deformation of the cupula and sinusoidal angular acceleration of the NBSC and the MBSC.

An angular acceleration perception experiment were carried out on the MBSC and NBSC models, and the results were shown in Fig. 8. As shown in Fig. 8a and Fig. 8b, when the two models were subjected to an impact oscillation, the angular accelerations of the two BSC model shells exhibited impact waveforms. The central point displacements at the two cupulas all reached their maximums rapidly and then oscillated around their equilibrium positions gradually decreasing until the static position finally. The oscillation waveform was the same as the theoretical waveform obtained from Eq. (37). It can be observed from the partially enlarged views that the cupula oscillation in the MBSC is more intense than that in the NBSC. This result suggested that the elastic coefficient of the spring in the mechanical model of the MBSC was greater than that of the NBSC, and the MBSC could store more energy than the NBSC. What's more, the displacement amplitudes of the central point of the two cupulas were linearly related to the angular acceleration amplitudes of the two shells, as shown in Fig. 8c. Similarly, the displacement at the central point of the cupula in the MBSC was smaller than that in the NBSC. These results verified the conclusion of Eq. (37).

It could be observed from Fig. 8d and Fig. 8e, when the two BSC models were subjected to a sinusoidal oscillation, the displacements at the central point of the two cupulas exhibited a sinusoidal waveform. The frequency of the displacements was the same as those of the two BSC model shells. In addition, the amplitudes at the central point of the two cupulas were linearly related to those of the angular acceleration of the two BSC model shells, as shown in Fig. 8f. When the two shells oscillated with the same amplitude, the displacement at the central point of the cupula in the MBSC was less than that in the NBSC. These results verified the conclusion of Eq. (24).

**Figure 9:**
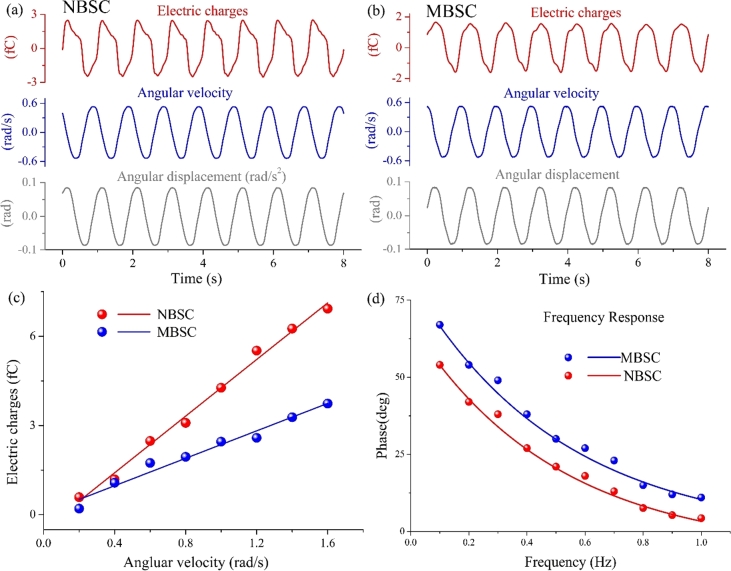
The experimental results of angular velocity and phase difference of the two models. (a) The results of the NBSC's sinusoidal rotating experiment. (b) The results of the MBSC's sinusoidal rotating experiment. (c) The relationship between the deformation of the cupula and sinusoidal angular velocity of the NBSC and the MBSC. (d) The phase difference between the cupulas and the shells of the two models.

When the two BSC models were subjected to a sinusoidal oscillation, their angular velocity exhibited a cosine waveform, as shown in Fig. 9a and 9b. The frequencies of angular displacement, angular velocity, and cupula deformation are the same. The amplitudes at the central point of the two cupulas were also linearly related to those of the angular velocity of the two models, as shown in Fig. 9c.

At the same time, the deformation of the cupula in the two models lagged behind the shells due to the inertia of the fluid and the elastic of the models. The phase difference between the cupulas and the shells were shown in Fig. 9d. As the frequency increases, the phase difference gradually decreases. It is consistent with the traditional biomechanical conclusions of human semicircular canals [48]. With the same rotational frequency, the phase difference of MBSC is larger than that of NBSC. It verified the mechanical models of the MBSC and the NBSC.

For simplicity, in the MBSC model, the bionic membrane semicircular canal is located in the middle of the shell. But actually, the membrane semicircular canal is located outside the center of the bony semicircular canal [47]. So in humans, the relative length of the membranous semicircular canals is longer than that in the MBSC, and it has a little more endolymph. In the mechanical model of the MBSC, *m* can be a little large. The relative amplitude at the central point of the cupula in humans could be a little larger than that in the MBSC. However, since the increase in endolymph was small, it had less effect on the experimental results.

Based on the experimental results of angular acceleration perception of the MBSC and NBSC, some deductions can be obtained as follows. When a human head swings, the cupula in the corresponding semicircular canal can produce concave and convex deformation. When the head is subjected to an impact swing, the cupula should deform quickly to a maximum, and then repeatedly attenuates the oscillation around the equilibrium position until it stops at a static position. When the head is subjected to an impact vibrating swing, the displacement at the central point of the corresponding cupula should also exhibit an impact waveform, its frequency should be the same as those of the swinging head, and its amplitude should be linear with the angular acceleration. When the head is subjected to a sinusoidal swing, the displacement at the central point of the cupula can also exhibit a sinusoidal waveform. Its frequency should be the same as that of the swinging head, and its amplitude should be linear with the angular velocity and acceleration. In a conclusion, the human can perceive the waveform, frequency, amplitude, and other related parameters of his swinging head through sensing the concave and convex deformation of the cupula.

Based on the mechanical models of the two BSCs, the relationship between the output electric charges of the SMPF and the angular acceleration of the two BSCs were established. The experimental results verified the mechanical models. Based on these results, it can be concluded that the membranous semicircular canal in humans not only has the function of isolating the perilymph and the endolymph but also can play a role as a spring in biomechanics. When the human head rotates, the elastic membranous semicircular canal can absorb a part of the kinetic energy caused by the endolymph and reduce the deformation of the cupula in the semicircular canal. When the human head stops rotating, the elastic membrane can release a part of the energy and drives the endolymph to return to its equilibrium position. In a word, the elastic membrane has a function of energy storage.

## 5 Conclusion

In this paper, an MBSC and an NBSC were designed and fabricated. An SMPF artificial hair sensor was fabricated to imitate the sensory hair cells in the human semicircular canal, the resin was used to imitate the hard shell of the human bone semicircular canal, silicone rubber was used to fabricate a bionic membranous semicircular canal and the cupula in the ampulla, and deionized water was used to imitate the endolymph and perilymph. The two BSCs could imitate not only the fine structure but also the function of the semicircular canal in the human body.

A BA was designed and fabricated based on the SMPF artificial hair sensor. The experimental results suggested that the SMPF sensor could accurately imitate the structure and perception function of the sensory hair cells in the human semicircular canal. Based on the BA, an MBSC and an NBSC were designed and fabricated, and their mechanical models were established. When the shells of MBSC and NBSC were together subjected to a sinusoidal swing or an impact swing, the mechanical models showed that the displacements at the central point of the cupula in the two BSCs also exhibited sinusoidal waveforms or impact waveforms. The mechanical models of the MBSC and NBSC were verified by the experiments using the electric charges of the SMPFs sensor. As a result, it was found that the semicircular canals can sense the angular acceleration of the human head. The endolymph had a function of liquid mass while the membranous semicircular canal had a function similar to a spring, which could store energy.

## Declarations

### Author contribution statement

Yixiang Bian: Conceived and designed the experiments.

Shien Lu, Zhi Wang, Yongbin Qin, Jialing Li: Performed the experiments; Contributed reagents, materials, analysis tools or data.

Guangming Guo, Junjie Gong: Conceived and designed the experiments; Analyzed and interpreted the data.

Yani Jiang: Conceived and designed the experiments; Analyzed and interpreted the data; Wrote the paper.

### Funding statement

Dr. Yixiang Bian was supported by 10.13039/501100001809National Natural Science Foundation of China [51775483].

### Data availability statement

Data will be made available on request.

### Declaration of interests statement

All authors declare that there are no conflicts of interest.

### Additional information

No additional information is available for this paper.
